# Supplementary material for: Stratification of Breast Cancer by Integrating Gene Expression Data and Clinical Variables
Source: Molecules. 2019 Feb 11;24(3):631. doi: 10.3390/molecules24030631 (PMC6385100; doi:10.3390/molecules24030631)
Supplement: Supplementary file 1 [file molecules-24-00631-s001.zip › molecules-428335-proofed-SM/Supplement File S1.pdf]

molecules

Supplement File S1. Clustering results based on mRMR and k-means clustering and parameter determination.

In mRMR feature (gene) selection, the number of top relevant genes *pool* and the number of finally selected, simultaneously nonredundant genes *k* for each clinical variable are determined experimentally. We set *k* = [50, 100, 150, 200] and *pool* = [1000, 2000, 3000] in our experiment and identified the optimal parameter *k* =100 and *pool* =2000 as the final parameter in our study based on the clustering result. The clustering outcomes under different parameters are given in Table S1, where "Gene number" denotes the number of union candidate genes selected for all clinical variables in our study, and "ARI with PAM50" was calculated by the adjustedRandIndex() function in R software to measure the agreement between mRMR subtypes with PAM50. The significant p values of association between subtypes and survival time/ recurrence time were transformed by -log10(-log10(SUR)/ -log10(RFS)). The bold content in the table denotes p values that are smaller than 0.05 (-log10(0.05)=1.30).

We can conclude from the table that when *pool*=2000, all subtypes under distinct k are significantly correlated with survival and recurrence; moreover, the agreement with PAM50 achieved best when *pool*=2000 and *k*=100. Finally, we chose *pool*=2000 and *k*=100 as the optimal parameters for further analysis.

Table S1. Clustering results based on mRMR and k-means clustering

mRMR	k	50	100	150	200
pool=1000	Gene number	556	1084	1589	2072
	ARI with PAM50	0.2741	0.3006	0.3199	0.3333
	-log10(SUR)	**9.17**	**7.77**	**7.63**	**5.37**
	-log10(RFS)	1.22	1.05	1.16	0.97
pool=2000	Gene number	552	1064	1558	2038
	ARI with PAM50	0.2782	**0.3337**	0.3169	0.3196
	-log10(SUR)	**7.71**	**5.04**	**5.02**	**5.82**
	-log10(RFS)	**1.55**	**1.79**	**1.87**	**1.61**
pool=3000	Gene number	550	1061	1542	2010
	ARI with PAM50	0.2734	0.3248	0.3072	0.3144
	-log10(SUR)	**5.41**	**5.54**	**4.89**	**3.92**
	-log10(RFS)	0.57	1.3	**1.67**	**1.61**
