# Supplementary material for: Stratification of Breast Cancer by Integrating Gene Expression Data and Clinical Variables
Source: Molecules. 2019 Feb 11;24(3):631. doi: 10.3390/molecules24030631 (PMC6385100; doi:10.3390/molecules24030631)
Supplement: Supplementary file 1 [file molecules-24-00631-s001.zip › molecules-428335-proofed-SM/Supplement File S2.pdf]

molecules

MDPI

Supplement File S2. Heat maps and PCAs for RNA-Seq of genes of subtypes based on mRMR and HV models.

In this study, we selected 1064 gene expressions of 1035 breast tumor samples that have maximum relevance with clinical variables and minimum redundancy themselves utilizing mRMR feature selection and obtained subtypes based on these mRMR-selected genes and k-means clustering. And we concluded that our subtypes are more significantly relevant to survival and recurrence compared to the prediction analysis of microarray 50 (PAM50) and highest variability (HV) methods, which use only expression data. The HV approach selected the top 2000 features (genes) showing the highest variability (largest standard deviation) to execute k-means clustering for breast stratification, which is widely used for extraction of the most informative features from gene expression data. Clusters of both mRMR and HV feature-screening methods exhibited almost the same moderate concordance with PAM50 calls, and RNA-seq heat maps showed that mRMR subtypes (Figure S1a) exhibited more diverse expression pattern compared to HV subtypes (Figure S1b).

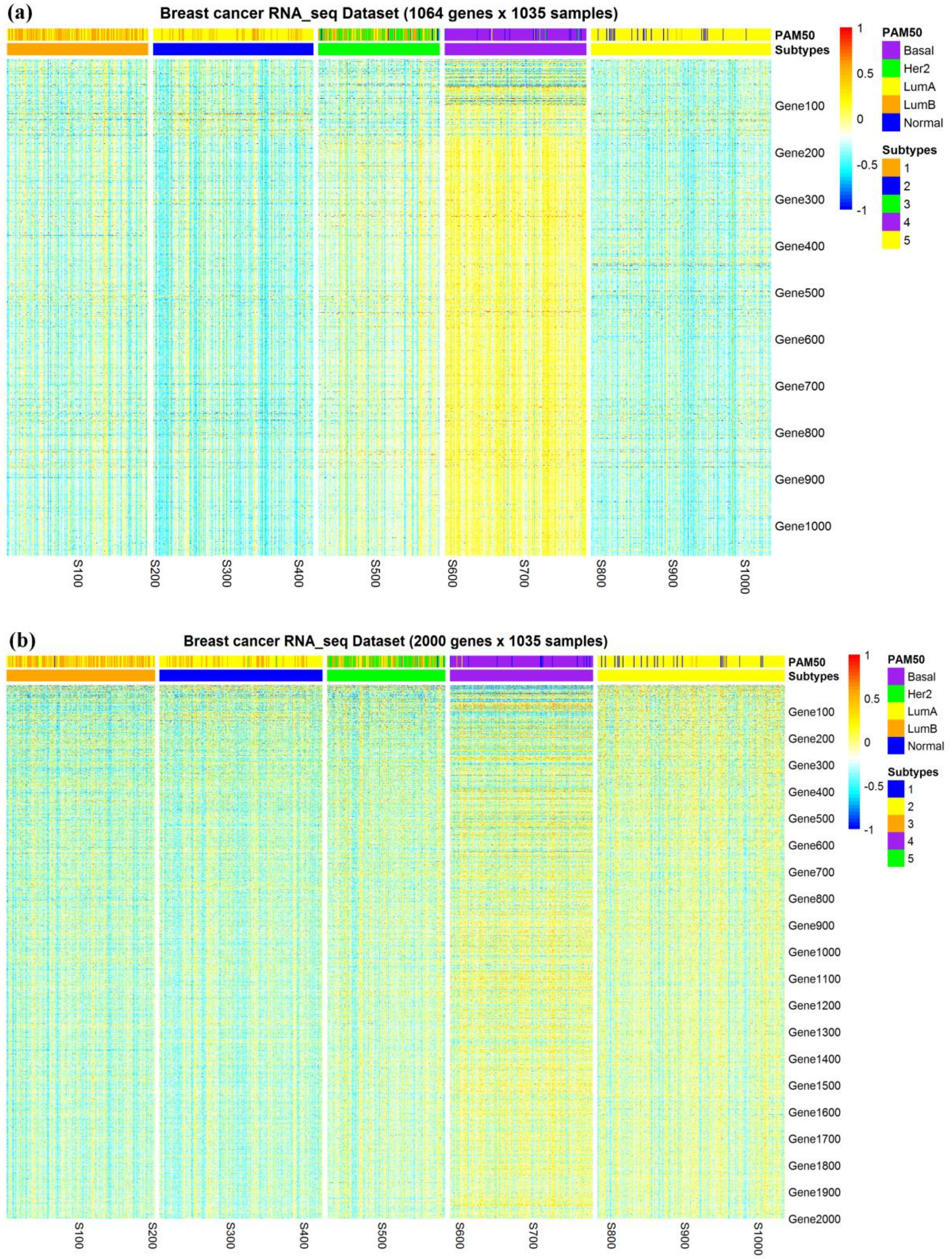

Figure S1. RNA-Seq pattern of clustering subtypes of 1035 breast tumor samples. **(a)** A partition based on 1064 mRMR-selected gene expressions and the k-means algorithm exhibited moderate agreement with PAM50 calls. **(b)** A partition based on top 2000 gene expressions selected by HV model and the k-means algorithm also exhibited moderate agreement with PAM50 calls.

Additionally, principal component analysis (PCA) were plotted to visualize the clustering distribution of the breast tumor subtypes in low-dimensional space based on RNA-Seq of genes. Through Figure S2, we found that based on three principal components of 1064 gene expressions selected by mRMR, five breast subtypes were more clearly separated by mRMR and HV models

compared with PAM50 subtypes (Figure S2a-c). Moreover, subtypes obtained by mRMR model based on 1064 mRMR-selected genes (Figure S2b) were more clearly separated than that obtained by HV model based on top 2000 HV-selected genes (Figure S2d).

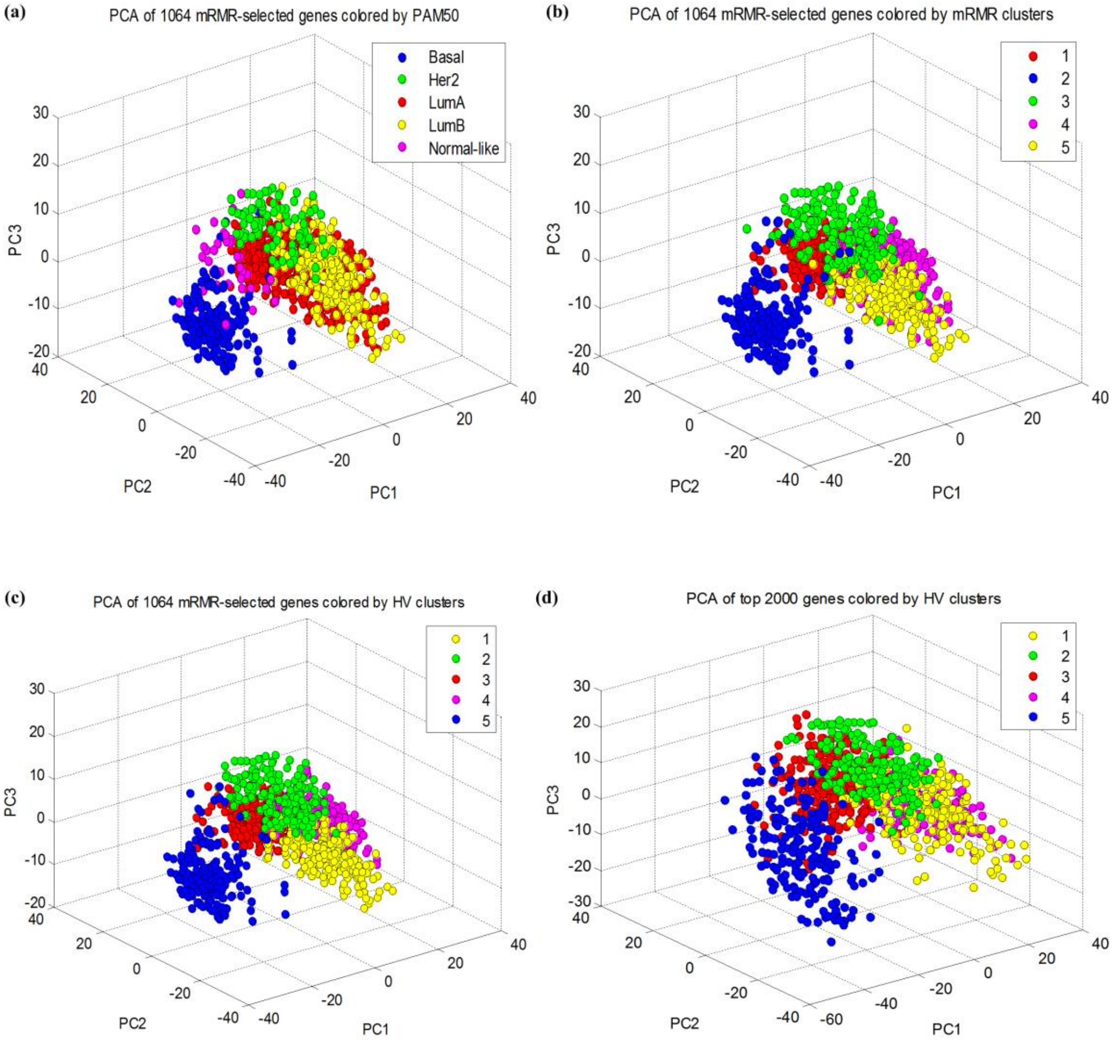

Figure S2. PCAs of 1035 breast tumor samples. **(a)** PCA based on 1064 mRMR-selected genes, colored by PAM50 labels. **(b)** PCA based on 1064 mRMR-selected genes, colored by mRMR clusters. **(c)** PCA based on 1064 mRMR-selected genes, colored by HV clusters. **(d)** PCA based on top 2000 HV-selected genes, colored by HV clusters.
